# Genetic diversity and structure of an endangered desert shrub and the implications for conservation

**DOI:** 10.1093/aobpla/plx016

**Published:** 2017-04-18

**Authors:** Zhihao Su, Bryce A. Richardson, Li Zhuo, Xiaolong Jiang, Wenjun Li, Xiaoshan Kang

**Affiliations:** 1 Key Laboratory of Biogeography and Bioresource in Arid Land, Xinjiang Institute of Ecology and Geography, Chinese Academy of Sciences, no. 818 South Beijing Road, Xinjiang, Urumqi 830011, China; 2 USDA Forest Service, Rocky Mountain Research Station, 735 North 500 East, Provo, UT 84606, USA; 3 Library, Xinjiang Normal University, Urumqi 830054, China; 4 Shanghai Chenshan Plant Science Research Center, Shanghai Chenshan Botanical Garden, Chinese Academy of Sciences, Shanghai 201602, China

**Keywords:** Conservation, gene flow, genetic drift, hydrochory, *Tamarix taklamakanensis*, Tarim Basin

## Abstract

Population genetic information can provide valuable insight for the conservation and management of threatened and endangered plant species. *Tamarix taklamakanensis* is an endangered shrub endemic to arid basins of northwestern China. This species serves to stabilize soils in this region, but has seen substantial loss in its abundance due to depletion of ground water. The populations of this species have become small and fragmented, warranting conservation. Seven microsatellite loci were used to assess the genetic diversity and structure of 15 populations in the Tarim Basin, China. Among populations, the expected heterozygosity and total gene diversity were both moderate (*H*_E_ = 0.392, *h*_T_ = 0.432), however the allelic diversity was low (*A* = 2.4). Eleven populations were detected to have experienced recent bottlenecks using Wilcoxon’s test and a model-shift test. Most populations of *T. taklamakanensis* in the centre of Tarim Basin showed low levels of genetic differentiation, but higher levels in geographically outlying populations. Genetic structure based on Bayesian assignment, the neighbour-joining network and principal coordinates analyses produced similar results, supporting five groups in the Tarim Basin. Gene flow was high among Bayesian groups based on historical gene flow estimated by private alleles. The genetic structure of *T. taklamakanensis* supports a pattern where gene flow principally occurs along river corridors through hydrochory of seeds and insect-mediated pollination. Populations upstream have contributed to a more diverse mixture of populations near the confluence of several rivers near the centre of Tarim Basin. This pattern of genetic structure could be influenced by the flow of water from different river systems. Conservation efforts should focus on fostering the regeneration of this species, maintaining genetic diversity and preserving the extant genetic structure. Conservation efforts are contingent upon maintaining ground water and streamflows in this arid basin.

## Teaser

Tamarix taklamakanensis is an endangered shrub endemic to arid basins of northwestern China. Based on seven microsatellite loci, we assessed the genetic diversity and structure of 15 populations in the Tarim Basin, China. We found that the total gene diversity was moderate, whereas the allelic diversity was low; we also found high levels of gene flow among populations of T. taklamakanensis in the center of Tarim Basin, but higher levels genetic differentiation in geographically outlying populations. Our study will provide useful information for conservation and restoration.

## Introduction

Fragmentation has been recognized as one of the factors increasing the vulnerability of many plant species (Lande 1998; Davies *et al.* 2001; Young and Clarke 2000). It reduces population sizes, increases spatial isolation among populations, changes the abundance and behavior of pollinators, and alters seed dispersal (Morris 1993; Kremen and Ricketts 2000; England *et al.* 2002; Frankham *et al.* 2002). Consequently, this may cause a loss of genetic diversity through genetic drift by increasing the genetic isolation and inbreeding (Ellstrand and Elam 1993; Frankham 1995; England *et al.* 2002). The loss of genetic diversity will in turn weaken the fitness of populations, increasing the vulnerability to extinction (Newman and Pilson 1997; Saccheri *et al.* 1998).

Habitat fragmentation can affect plant species differently depending on their life history traits. The erosion of genetic diversity caused by habitat fragmentation depends on multiple life traits, including population size, distance between populations, time since isolation, seed and pollen dispersal distance, and generation time (Frankham *et al.* 2002; Juan *et al.* 2004; Yao *et al.* 2007). Thus, genetic responses to habitat fragmentation are not universal. Some studies have shown fragmented species have reduced genetic diversity and high genetic differentiation due to impediments to gene flow and drift (Young and Clarke 2000; Fuchs *et al.* 2003; Kang *et al.* 2008); whereas in other species, high genetic variation (Young *et al.* 1993; Jacquemyn *et al.* 2004) and low genetic differentiation (Aldrich *et al.* 1998; Hogbin *et al.* 1998; Bacles *et al.* 2005) have been maintained.

For endangered plant species, information on genetic diversity and structure is indispensable before conservation and restoration management decisions are undertaken (Segarra-Moragues *et al.* 2005; Friar *et al.* 2001). In this respect, neutral markers provide information on genetic structure, gene flow, and genetic drift among and within populations, giving insight into past demographic events (Milligan *et al.* 1994; Galeuchet *et al.* 2005; Segarra-Moragues *et al.* 2005; Richardson and Meyer 2012) and barriers that affect gene flow (Petit *et al.* 2002). Population genetic information can help mitigate extinction risks by discerning intraspecific management units, and preventing inbreeding or outbreeding depression for *in situ* and *ex situ* conservation (Frankham *et al.* 2002).

*Tamarix taklamakanensis* is a large salt- and drought-tolerant shrub found principally in Taklamakan Desert within the Tarim Basin (Li 1990). Based on research of other *Tamarix* species, *T. taklamakanensis* is likely monoecious and insect pollinated (Wang *et al.* 2005; Chen and Zhao 2015). Like other species in this genus, *T. taklamakanensis*’ ability to persist in these arid conditions is contingent on utilizing deep soil water (He *et al.* 2014). This species is ground water dependent, occuring primarily along ephemeral waterways and lowland areas in the basin. It is more tolerant to salinity, high temperature and wind compared with the other species of *Tamarix* in China (Li 1990). Thus, it is an important shrub for soil stabilization in northwestern China (Liu 1987; Li 1990). However, its habitats have been highly fragmented and natural regeneration has been restricted over the past several decades. The loss of *T. taklamakanensis* is believed to be caused by ground water depletion by either agriculture diversion or extraction from wells (Liu *et al.* 1996; Yang *et al.* 2013). However, intensified climate drought could also be a factor. It has been listed as endangered in the China Species Red List due to its drastic decline (Fu 1992).

Genetic information of *T. taklamakanensis* is essential for its conservation and restoration. Today, no population genetic information exists for this species. Our goals are to better understand the genetic diversity and structure in this species and to infer potential barriers and corridors for gene flow. Here, we utilize seven polymorphic microsatellite loci to addressed the following questions: (i) What is the level of genetic diversity among populations of *T. taklamakanensis*? (ii) Is there significant genetic differentiation among populations? (iii) What evolutionary factors influence genetic diversity and genetic structure? These data will be helpful to infer the dominant evolutionary forces responsible for the observed genetic patterns, and will be of importance to devise ongoing conservation and management strategies for *T. taklamakanensis*.

## Methods

### Sample collection

**Figure 1:**
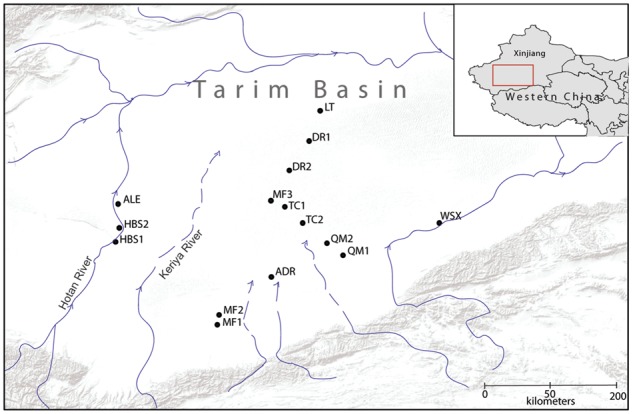
Geographic location of *T. taklamakanensis* sampled populations in northwestern China. Populations correspond to those in Table 1. Blue lines indicate river systems in the Tarim Basin and arrows show the direction of water flow.

In 2014, field samples were collected of *T. taklamakanensis* within the Tarim Basin. A total of 12–16 individuals were sampled at each of fifteen populations. GPS coordinates were recorded for each population (Fig. 1; Table 1). In total, 216 individuals of *T. taklamakanensis* were included in the analysis. Fresh leaves and stems were dried in silica gel and stored at 4 °C in preparation for DNA extractions.

**Table 1:** Details of sample locations and sample size for fifteen populations of *T. taklamakanensis*.

Population	Code	Sample size	Latitude	Longitude	Altitude (m)
Hongbaishan-1	HBS1	15	80.93	38.39	1175
Hongbaishan-2	HBS2	15	80.98	38.59	1156
Alaer	ALE	14	80.93	38.93	1136
Luntai	LT	12	84.34	40.26	924
Desert road-1	DR1	13	84.18	39.83	982
Desert road-2	DR2	15	83.87	39.41	1037
Mingfeng-3	MF3	15	83.58	38.98	1089
Tarim centre-1	TC1	16	83.83	38.89	1101
Tarim centre-2	TC2	15	84.16	38.66	1134
Qiemo-2	QM2	15	84.60	38.37	1168
Qiemo-1	QM1	15	84.89	38.20	1212
Andier	ADR	12	83.67	37.89	1269
Mingfeng-2	MF2	15	82.81	37.35	1269
Mingfeng-1	MF1	15	82.79	37.21	1269
Washixia	WSX	14	86.53	38.66	1214

### DNA extraction and microsatellite amplification

Total genomic DNA was extracted using a CTAB method (Doyle and Doyle 1987) from about 15 mg of dry-ground leaf and stem tissue. Polymerase chain reaction (PCR) was performed on 25 ng of genomic DNA in 25 µL reaction volumes. Reactions included 10 mM Tris-HCl, 50 mM KCl, 1.5 mM MgCl_2_, 0.2 mM dNTPs, 0.5 U Taq DNA polymerase and 5 µM of each primer. Amplification was conducted using a Biorad T100 thermocycler (Biorad) and using the protocol reported by Terzoli *et al.* (2013). Microsatellite loci were targeted using the primers described in Terzoli *et al.* (2013). To test the microsatellite primers on *T. taklamakanensis*, PCR was conducted with 30 samples from 6 populations. PCR products were screened by gel electrophoresis using 2 % agarose gel that was run for 2 h. Primer sets that produced bands of the expected length (Terzoli *et al.* 2013) were used for genotyping.

### Genotyping and data analysis

Seven microsatellite loci produced consistent PCR products near the expected size range and were separated by capillary electrophoresis, ABI 3730xl (Applied Biosystems) at the University of Wisconsin DNA-sequencing facility. Allele lengths were analysed with Geneious v7.0 using the package Plugin (Kearse *et al.* 2012), using Gene-flo 625 (Chimerx) as the internal lane standard.

Genetic diversity index, including the number of alleles per locus corrected for the sample size (A), Nei’s gene diversity index (h), observed heterozygosity (*H*_O_) and unbiased expected heterozygosity (*H*_E_) within each locus and population were calculated using GenAlEx 6.5 software (Peakall and Smouse 2006, 2012). Heterozygote deficiency within populations (*F*_IS_) at different significance levels (*P* = 0.05 and 0.001) and *F*-statistics value (*F*_IS_, *F*_IT_, and *F*_ST_) within each locus over all populations were calculated with FSTAT 1.2 (Goudet 1995). Deviation from Hardy-Weinberg expectations and linkage disequilibrium in pairs of microsatellites loci were tested with GENEPOP 3.4 (Raymond and Rousset 1995), using 1000 allelic permutations among individuals and 0.05 (*P*-value) for the significance level. Null alleles were tested with MICRO-CHECKER 2.2 (Van Oosterhout *et al.* 2004). Bonferroni-type correction was applied for all tests to estimate significance (Rice 1989).

Populations that have experienced recent bottlenecks generally exhibit significant excess of heterozygosity, indicating departure from mutation-drift equilibrium. Tests implemented in the programme BOTTLENECK 1.2.02 (Piry *et al.* 1999) were conducted under the infinite allele model (IAM), the stepwise mutation model (SMM) and the two phase model (TPM), with 10 000 replicates performed. The Wilcoxon sign-rank test suggested by Cornuet and Luikart (1996) was used to estimate the significance level. In addition, tests for shifted or normal L-shaped distribution of allele frequencies were also performed (Luikart *et al.* 1998).

Migration-drift equilibrium (gene flow model vs drift model) was tested with the software 2MOD, estimating the relative likelihoods of fthe two models using an Markov chain Monte Carlo (MCMC) procedure as described in Ciofi *et al.* (1999). The procedure was performed with 100 000 iterations, and the first 10 % of points in the output were dropped to avoid dependence on initial starting values.

To assess the partitioning of total genetic variation among and within populations, analysis of molecular variance (AMOVA) was implemented in ARLEQUIN v.3.01 (Excoffier *et al.* 1992), using 1000 permutations. Pairwise population differentiation measures (*F*_ST_) were calculated as the variance components (Wright 1965). To illustrate relationships among the populations, the *F*_ST_ matrix was used to construct a neighbour-joining (NJ) network in MEGA 6.0 (Tamura *et al.* 2013). The genetic distance matrix (*F*_ST_) was also used to perform principal coordinate (PCO) analysis, implemented in GenAlEx 6.5 (Peakall and Smouse 2006). Further examination of the population structure was conducted using a Bayesian approach that assigns initially sampled individuals into inferred groups, implemented in STRUCTURE 2.2 (Pritchard *et al.* 2007). To calculate the optimal number of genetically distinct groups (*K*), we first simulated a total of 10 000 MCMC iterations for the burn-in period, followed by a run length of 10 000 iterations. For each value of *K* (*K* = 2–10), three independent runs were performed to assure convergence and homogeneity among runs. We used deltaK to select the best *K*. Each run yielded a log likelihood value, Ln Pr (X/K), which had a corresponding deltaK. The highest Ln Pr(X/K) corresponded to the highest deltaK, and the maximum was chosen to determine the optimal number of genetically distinct clusters. DeltaK was calculated in Structure Harvester (http://taylor0.biology.ucla.edu/structureHarvester/). The probabilities of ancestor assignment were calculated for each pre-defined population (Pritchard *et al.* 2000). Based on the structure results, populations were grouped and hereafter referred to as groups. Genetic diversity index, including the number of alleles per locus corrected for the sample size (*A*), observed heterozygosity (*H*_O_) and unbiased expected heterozygosity (*H*_E_) corrected for the sample size, were calculated within the groups.

To examine whether the genetic distance have a significant relationship with the geographical distance, Mantel test was performed using the programme IBD v.1.52 (Bohonak 2002). Pairwise estimates of *F*_ST_, representing the genetic distances between populations, were calculated in GenAlEx 6.5, and the geographic distances between locations were calculated in GEODIS 2.5 (Posada *et al.* 2000). Geographic distances were first natural-log transformed when calculating the correlation index. The significance test was based on 10 000 permutations.

## Results

### Microsatellite diversity

A total of 30 alleles were detected among seven microsatellite loci from the 216 individuals of *T. taklamakanensis*. The mean number of alleles was 4.3, ranging from 2 at locus Th1239 and Th1286 to 8 at locus Th2287. The mean number of different alleles per population was 2.4, ranging from 1.7 in population WSX to 3.1 in population TC1. Within-population diversity (h_S_) was 0.4, total gene diversity (*h*_T_) was 0.432. The mean expected heterozygosity (*H*_E_) per locus was 0.392 (Table 2), ranging from 0.03 at locus Th1239 to 0.541 at locus Th1420, and ranging from 0.226 in population WSX to 0.473 in population TC1. The mean observed heterozygosity (*H*_O_) per locus was 0.573 (Table 2), ranging from 0.022 at locus Th1239 to 0.866 at locus Th669, and ranging from 0.39 in population QM1 to 0.838 in population MF1. The inbreeding level of each population (*F*_IS_) ranged from −0.094 to −0.913 (Table 2). No populations deviated from HWE, and no linkage disequilibrium was detected at between loci.

**Table 2:** Genetic diversity in populations and groups of *T. taklamakanensis*. Listed below are populations followed by assignment of groups defined by the programme structure (Pritchard et al. 2007).

Population	*n*	*A*	*H*_O_	*H*_E_	*F*_IS_
HBS1	15	2.3	0.560	0.380	–0.445
HBS2	15	2.4	0.593	0.398	–0.464
ALE	14	2.6	0.510	0.361	–0.383
LT	12	2.3	0.625	0.445	–0.365
DR1	13	2.4	0.474	0.357	–0.289
DR2	15	2.7	0.571	0.443	–0.258
MF3	15	2.9	0.543	0.430	–0.229
TC1	16	3.1	0.679	0.473	–0.408
TC2	15	2.3	0.657	0.408	–0.588
QM2	15	2.4	0.467	0.350	–0.304
QM1	15	2.6	0.390	0.346	–0.094
ADR	12	2.1	0.485	0.383	–0.222
MF2	15	2.0	0.800	0.444	–0.787
MF1	15	2.1	0.838	0.437	–0.913
WSX	14	1.7	0.398	0.226	–0.742
Average	14.21	2.4	0.573	0.392	
Group 1	15	2.3	0.560	0.380	–0.445
Group 2	30	2.3	0.819	0.442	–0.849
Group 3	14	1.7	0.398	0.226	–0.742
Group 4 and 5	157	4.3	0.548	0.421	–0.299

### Mutation-drift equilibrium

Using Wilcoxon’s test, 10 populations (HBS1, LT, DR1, DR2, MF3, TC1, TC2, ADR, MF2, MF1) showed a significant deviation from drift-mutation equilibrium under the assumption of IAM model, three populations (TC2, ADR, MF1) under the assumption of SMM model, and seven populations (HBS1, LT, DR2, TC2, ADR, MF2, MF1) under the assumption of TPM model. In addition, seven populations (HBS1, DR1, DR2, TC2, MF2, MF1, WSX) showed a shifted pattern in allele frequency distribution with mode-shift test (Table 3). These results suggested the occurrence of historical demographic bottlenecks in most populations of *T. taklamakanensis*.

**Table 3:** Tests for mutation-drift equilibrium and mode shift using BOTTLENECK.

Population	IAM	SMM	TPM	Mode shift
HBS1	0.05*	0.08	0.05*	Shift
HBS2	0.15	0.47	0.29	Normal
ALE	0.28	0.66	0.42	Normal
LT	0.02*	0.22	0.04*	Normal
DR1	0.03*	0.34	0.23	Shift
DR2	0.02*	0.28	0.05*	Shift
MF3	0.04*	0.50	0.22	Normal
TC1	0.05*	0.59	0.34	Normal
TC2	0.01**	0.04*	0.04*	Shift
QM2	0.29	0.77	0.71	Normal
QM1	0.29	0.81	0.53	Normal
ADR	0.02*	0.05*	0.02*	Normal
MF2	0.01**	0.15	0.01**	Shift
MF1	0.01**	0.02**	0.02**	Shift
WSX	0.09	0.16	0.09	Shift

Wilcoxon sign-rank test was used to estimate the significance level. IAM, the infinite allele model; SMM, stepwise mutation model; TPM, the two phase model; shift, shifted L-shaped distribution of allele frequencies; normal, normal L-shaped distribution of allele frequencies;

* represent significant at *P* < 0.05;

** represent significant at *P* < 0.01.

The migration-drift equilibrium analysis revealed a genetic drift model in *T. taklamakanensis*, with slightly higher likelihood than that of gene flow model (*P* (gene drift) = 0.51, Bayes factor = 1.03), indicating the presence of genetic drift but generally ample gene flow within the species. Sufficient gene flow was also supported by low genetic differentiation measures (*F*_ST_) in most pairs of the populations, discussed below.

### Population genetic structure

The majority of total genetic variation was found to occur within populations (79.00 %, *P* < 0.001). However, a moderate amount of the variation was found to occur among populations (21 %, *P* < 0.001; Table 4), and this variation was correlated with geographic distance. A significant linear relationship between genetic distances and transformed geographical distances was shown by a Mantel test (*r* = 0.486, *P* = 0.0002).

**Table 4:** Results of AMOVA for *T. taklamakanensis*.

Source of variation	d.f.	Sum of squares	Variance components	Percentage of variation	*P*
Among populations	14	124.305	*V*_a_ = 0.488	21	*P* < 0.001
Within populations	201	372.936	*V*_b_ = 1.855	79	*P* < 0.001
Total	215	497.241	2.343		

**Figure 2:**
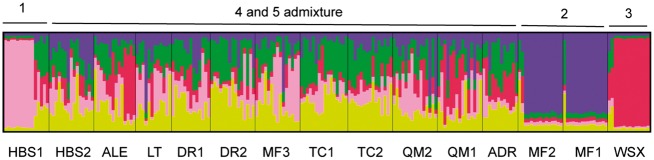
A barplot of individuals from fifteen populations of *T. taklamakanensis* using the programme STRUCTURE. Five inferred groups were represented by five colors (red, green, blue purple, pink, yellow). Each bar represents an individual with assignment probabilities to each group. The labels below the barplot refer to the population code in Table 1. The labels above the barplot represent how the populations are associated with the inferred groups.

**Figure 3:**
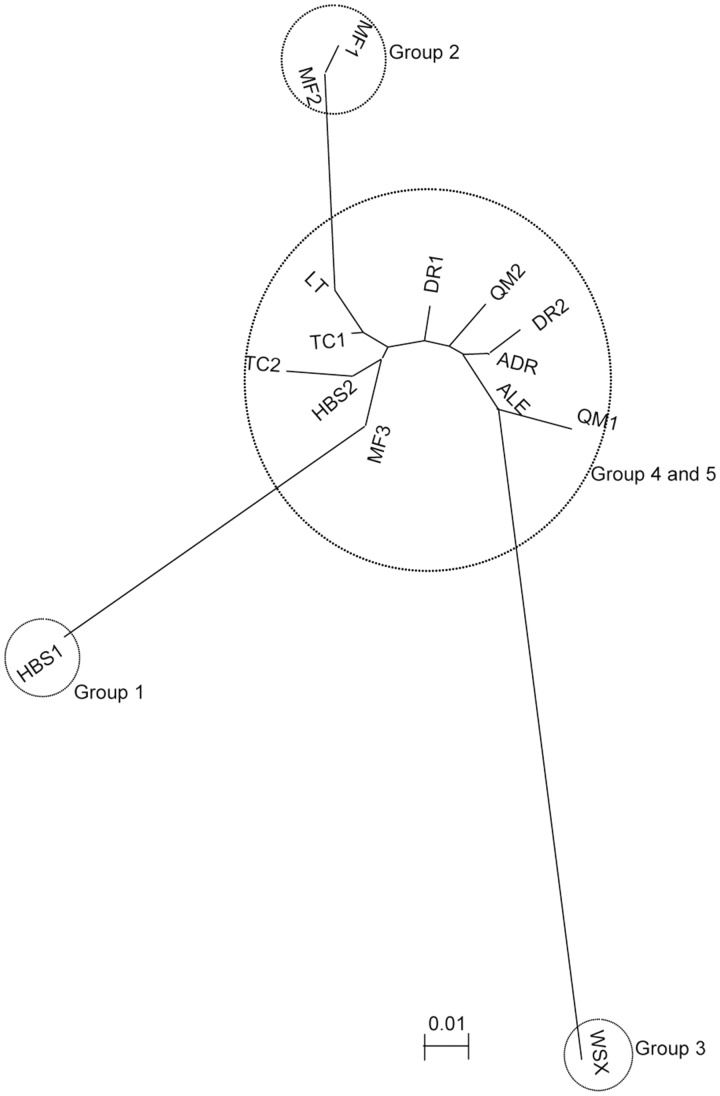
NJ dendrogram of 15 populations of *T. taklamakanensis* constructed using a *F*_ST_ matrix [**see Supporting Information—**Table S2].

**Figure 4:**
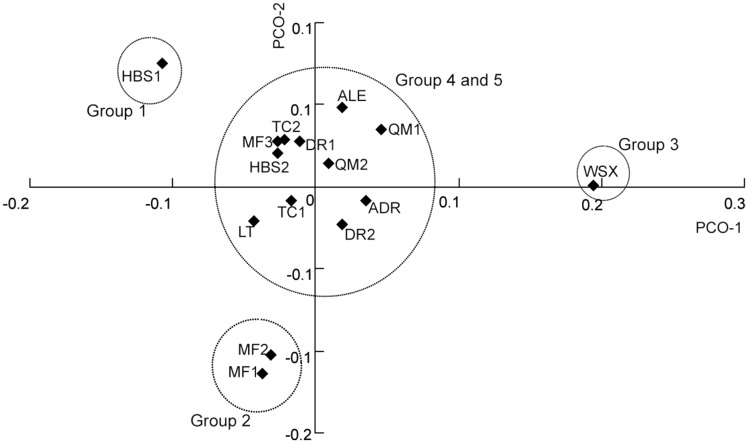
Plot of the first two coordinates based on pairwise population differentiation (*F*_ST_) matrix of *T. taklamakanensis*. PCO-1 and PCO-2 explain 39.9 and 24.6 % of the variation, respectively.

Genetic structure was assessed without *a priori* assumptions of population number (*K*) using STRUCTURE. The analysis indicated five inferred genetically distinct groups in *T. taklamakanensis*. At *K* = 5, two populations HBS1 and WSX were largely assigned to their own unique groups with moderately high assignment probabilities of 0.649 (HBS1) and 0.816 (WSX). Individuals from populations MF1 and MF2 were assigned into a group (Group 2) with probabilities of 0.765 and 0.724, respectively **[see Supporting Information—Table S1]**. The remaining 11 populations were assigned to admixed groups 4 and 5 (Fig. 2). The results were also consistent with the NJ network (Fig. 3) and PCO plots (Fig. 4). In the NJ network, populations WSX, HBS1, MF1 and MF2 were distinct from the other 11 populations, which grouped together. In the PCO diagram, the first and the second axis respectively accounted for 39.9 and 24.6 % of the total variation, and populations were separated into four clusters, including WSX, HBS1, MF1 and MF2, and remaining populations (Fig. 4). These results demonstrated high levels of gene flow among populations of *T. taklamakanensis* in the centre of the basin, and suggest populations are more structured around the periphery of Tarim Basin, consistent with results of the migration-drift equilibrium analysis. The allele number ranged from 1.7 in group 3 to 4.3 in groups 4 and 5, the expected heterozygosity (*H*_E_) ranged from 0.226 in group 3 to 0.442 in group 2, and the observed heterozygosity (*H*_O_) ranged from 0.398 in group 3 to 0.819 in group 2.

## Discussion

### Genetic diversity

*T. taklamakanensis* has maintained a moderate level of genetic diversity, despite the decline in abundance and increasing fragmentation seen in this species over the past few decades (Liu *et al.* 1996; Yang *et al.* 2013). When compared with closely related species within the Tamaricaceae distributed in China, the microsatellite heterozygosity of *T. taklamakanensis* (*H*_E_ = 0.392) was lower than that of *T. chinensis* (*H*_E_ = 0.49; Zhu *et al.* 2016), and higher than that of *Reaumuria trigyna* (mean *H*_E_ = 0.25; Qi 2015). The total gene diversity (*h*_T_ = 0.432) was higher than that *Reaumuria soongorica* (*h*_T_ = 0.312) (Qian *et al.* 2008). The maintenance of genetic diversity could be explained by the life cycle and breeding system of *T. taklamakanensis*. It has been demonstrated that long-lived and outcrossing species are capable of maintaining higher levels of genetic diversity compared with annuals or short-lived periennials (Loveless and Hamrick 1984; Hamrick and Godt 1989; Austerlitz *et al.* 2000). *T. taklamakanensis* is known to live several decades (Yuan *et al.* 2011), and based on congeners (Chen and Zhao 2015; Wang *et al.* 2005), this species is likely insect pollinated with facultative outcrossing. Therefore, these life history traits may provide some degree of buffering to genetic erosion caused by fragmentation and loss in *T. taklamakanensis*.

Although heterozygosity suggests moderate levels of genetic diversity, measures of allelic diversity provide somewhat different results. The observed allelic diversity in populations of *T. taklamakanensis* was low (2.4), compared with those reported in some tree species, such as *Changiostyrax dolichocarpa* (*A* = 4.4–6.1; Yao *et al.* 2007), *Grevillea macleayana* (*A* = 3.2– 4.2; England *et al.* 2002), and *Santalum austrocaledonicum* (*A* = 2–16; Bottin *et al.* 2005). The Bayesian analysis supported groups 4 and 5 maintained moderate allelic diversity (*A* = 4.3), whereas the other three groups, located around the perimeter of the Tarim Basin, all had low levels of allelic diversity (Table 2). Thus, the observed allelic diversity of *T. taklamakanensis* was generally low. Furthermore, allelic diversity is sensitive to the effect of genetic erosion caused by recent demographic bottlenecks (Nei *et al.* 1975; Spencer *et al.* 2000). Our analysis suggests 11 of the 15 populations were affected by recent bottlenecks (Table 3), and their *F*_IS_values were all large and negative, implying reduced size of these populations (Keller and Waller 2002; Stoeckel *et al.* 2006), which could have contributed to the low allelic diversity. The reduced size of populations during the bottleneck would increase genetic drift and lead to the loss of allelic diversity (Gaudeul *et al.* 2000).

### Population differentiation, gene flow and hydrochory

Overall, population genetic structure in *T. taklamakanensis* was moderate with 21 % of the variation existing among populations (Table 4). However, *F*_ST_ values varied considerably among population pairwise comparisons [e.g. *F*_ST_ = 0–0.37, **see Supporting Information—Table S2**]. Bayesian groups 1 (HBS1), 2 (MF1 and MF2) and 3 (WSX), which exhibited higher assignment probabilities and greater genetic structure (Fig. 3), all have common landscape attributes: (i) these groups are all upstream of populations located in the centre of the basin, and (ii) these groups are located on different river drainages (Fig. 1). Groups 1–3 are in contrast to groups 4 and 5. Groups 4 and 5, located in the centre of the basin at lower elevations and closer to the confluence of several river drainages (Fig. 1), have substantially more admixture (i.e. lower assignment probabilities) and greater genetic diversity (Table 2). Groups 4 and 5 may reflect gene flow from other outlying and distinct populations not sampled in this study.

A hypothesis that supports the genetic patterns described earlier is hydrochory, the dissemination of propagules via water. *T. taklamakanensis* seeds are minute and could travel long distances by wind and water (Yang and Gaskin 2007). However, *Tamarix* species are facultative phreatophytes (Tomaso and Tomaso 1998). Under such arid conditions in the Tarim Basin, *T. taklamakanensis* is restricted to areas with ground water, typically river drainages, such as the Hotan River and other small river systems. The geographic distances between river corridors are typically far (> 60 km), and likely beyond the flight range of pollinators or seed dispersal. Thus, given that the areas surrounding river drainages are barren, gene flow could be restricted to river corridors. Hydrochory has been shown to be an important dispersal mechanism of plant propagules (Nilsson *et al.* 2010). Gene flow in hydrocory is largely unidirectional with the flow of water. Consequently, hydrocory produces higher genetic diversity in downstream populations and greater genetic differentiation among populations in separate tributaries (Love *et al.* 2013).

### Conservation implications

Our study provides valuable insight into the conservation and management of *T. taklamakanensis.* The detected population bottlenecks (Table 3) and observed low allelic diversity within populations (Table 2) suggest this species is at a threshold where further habitat decline (i.e. groundwater depletion) could erode genetic diversity. *In situ* conservation is needed and should be performed to: (i) mitigate the loss of genetic diversity and (ii) preserve the extant genetic structure. Priority should be given to disjunct populations with lower allelic diversity, in particular population in groups 1 and 4. *In situ* conservation and restoration efforts of *T*. *taklamakanesis* are dependent on conserving the groundwater in the basin. Unless water consumption from river diversion and well drilling is curbed, work from conservation efforts will likely be undermined. Given these circumstances, *ex situ* conservation should also be considered. A germplasm nursery of *T. taklamakanensis* has been established in Turpan Botany Garden. However, this *ex situ* nursery is lacking the genetic information revealed in our study, and currently captures only a small part of the total genetic variation. Given that severe drought could devastate entire populations of this species or climate change may necessitate assisted migration, comprehensive germplasm storage would be essential for population recovery.

## Sources of Funding

This research was supported by grants from National Natural Science Foundation of China (31400561), the Western Doctoral Project (XBBS201306, 2016-QNXZ-B-16, XBBS201310) of Xinjiang Institute of Ecology and Geography, Chinese Academy of Sciences.

## Contributions by the Authors

Z.H.S. wrote the article; B.A.R. designed the experiment and revised the article. All authors contributed to execution of the experiment.

## Conflict of Interest Statement

None declared.

## Supporting Information

The following additional information is available in the online version of this article —

 **Table S1.** Pairwise estimated values of *F*_ST_ (below diagonal) among the populations of *T. taklamakanensis* and significance (above diagonal, *P < 0.05; **P < 0.001) tested using the infinite alleles model.

 **Table S2.** The assignment probabilities of membership of fifteen predefined populations in each of the five clusters inferred by STUCTURE.
